# Pitfalls in genetic testing: a case of a SNP in primer‐annealing region leading to allele dropout in *BRCA1*

**DOI:** 10.1002/mgg3.295

**Published:** 2017-05-11

**Authors:** Felipe Carneiro Silva, Giovana Tardin Torrezan, Rafael Canfield Brianese, Raquel Stabellini, Dirce Maria Carraro

**Affiliations:** ^1^ Laboratory of Genomics and Molecular Biology International Research Center AC Camargo Cancer Center São Paulo Brazil; ^2^ Genomic Diagnostics Laboratory Anatomic Pathologic Department AC Camargo Cancer Center São Paulo Brazil

**Keywords:** Allele dropout, *BRCA1*, genetic testing, misdiagnosis

## Abstract

**Background:**

Hereditary breast and ovarian cancer is characterized by mutations in *BRCA1* or *BRCA2* genes and PCR‐based screening techniques, such as capillary sequencing and next‐generation sequencing (NGS), are considered gold standard methods for detection of pathogenic mutations in these genes. Single‐nucleotide polymorphisms (SNPs) constitute a vast source of variation in the human genome and represent a risk for misdiagnosis in genetic testing, since the presence of a SNP in primer‐annealing sites may cause false negative results due to allele dropout. However, few reports are available and the frequency of this phenomenon in diagnostic assays remains unknown.

**Methods and Results:**

In this article, we investigated the causes of a false negative capillary sequencing result in *BRCA1* involving a mother‐daughter dyad. Using several molecular strategies, including different DNA polymerases, primer redesign, allele‐specific PCR and NGS, we established that the initial misdiagnosis was caused by a SNP located in the primer‐annealing region, leading to allele dropout of the mutated allele.

**Conclusion:**

Assuming that this problem can also occur in any PCR‐based method that are widely used in diagnostic settings, the clinical report presented here draws attention for one of the limitations of genetic testing in general, for which medical and laboratory communities need to be aware.

## Background

Hereditary breast and ovarian cancer (HBOC – MIM 604370, MIM 612555) is characterized by pathogenic mutations in *BRCA1* (MIM 113705) or *BRCA2* (MIM 600185) genes. As a result of a widely diverse genetic background of populations, the complete screening of pathogenic germline mutations in both genes has been crucial for molecularly diagnosing affected patients (Carraro et al. 2013; Silva et al. 2014; Kast et al. 2016). For decades, PCR‐based capillary sequencing has been the gold standard method for DNA sequencing and mutation analysis. As all PCR‐based techniques, the efficiency of fragment amplification is mainly dependent on primer specificity in annealing the template sequence and one single nucleotide mismatch between primer and template can influence the amplification yield. The preferential amplification of one of the alleles can lead to a biased final amplicon composition and an incorrect genotype call, a phenomenon known as allele dropout (Wu et al. 2009; Gray et al. 2015).

In this article, we described and investigated the causes of a false negative capillary DNA sequencing result in *BRCA1* gene. Using several molecular biology strategies, such as different DNA polymerases, primer redesign, allele‐specific PCR and NGS, we confirmed that the initial misdiagnosis was caused by the presence of a SNP in the primer‐annealing region that impairs the mutated allele amplification, causing an allele dropout.

## Patients and methods

### Ethical compliance

The study was performed in compliance with the Declaration of Helsinki and was approved by the ethics committee of A. C. Camargo Cancer Center (EC 37/16). Both patients signed the written informed consent applied for genetic tests performed in A.C. Camargo Laboratory of Genomic Diagnostic.

### Case presentation

A breast cancer patient (hereafter denoted as *mother*) was referred for specific mutation analysis in *BRCA1* gene (c.5074+2T>C) by the Oncogenetic Department of A. C. Camargo Cancer Center. Peripheral blood was collected and sent to the Genomics Diagnostic Laboratory of the same institution. Routine genetic testing for *BRCA1* exon 17 using capillary sequencing was negative for the specific mutation.

Due to the previously positive test result for this mutation in the patient's daughter, the case was investigated in depth. Family history was assessed through personal report and included an affected daughter, who developed triple negative breast cancer at age 42 (carrier of the *BRCA1* c.5074+2T>C [NM_007294.3] mutation and whose genetic testing was also performed in the A. C. Camargo Genomics Diagnostic Laboratory); and an affected mother, with breast cancer at age 53 (no genetic test available). The patient presented a triple negative breast cancer at age 60.

### Mutation analysis

Routine genetic testing in *BRCA1/2* genes and specific for the *BRCA1* exon 17 were performed using capillary sequencing with primers designed for the coding exons and the intron‐exons limits, as described previously (Carraro et al. 2013; Silva et al. 2014). Additionally, PCR amplification and sequencing of *BRCA1* exon 17 was repeated several times in the mother sample, using four different DNA polymerases (GoTaq/Promega, Kapa/Kapa Biosystems, Platinum Taq and Platinum Taq High Fidelity/Invitrogen) and multiple PCR conditions. A novel primer pair for *BRCA1* exon 17 nonoverlapping with the routine pair was designed to confirm the presence of the c.5074+2T>C mutation in the mother. Allele‐specific PCR (AS‐PCR) followed by capillary sequencing was performed to confirm the haplotype phase of the c.5074+2T>C mutation and the rs8176233 polymorphism. Primers were designed so that the 3′ base was specific complementary for the reference (A) or the polymorphic nucleotide (G) of SNP rs8176233. Targeted NGS was performed for *BRCA1/2* genes (coding regions and intron‐exons boundaries) as described in details in the Supporting Information. PCR conditions and primers are available upon request.

## Results

A genetic testing for a specific pathogenic *BRCA1* variant (c.5074+2T>C) was performed on one breast cancer patient (mother). The capillary sequencing analysis revealed to be negative for this mutation. The patient's daughter presented a previously positive result for the complete genetic testing of the *BRCA1* and *BRCA2* genes, with the detection of a heterozygous splice site variant in *BRCA1* intron 17 (c.5074+2T>C; rs80358089), which is classified as pathogenic according to ClinVar and BIC databases. Due to the inconsistency of the two testing results, facing the relationship and family history of the patients, we initiated a deep investigation to understand why the mother showed an unexpected negative result when screened using capillary sequencing, which is considered the gold standard method for mutation detection.

**Figure 1:**
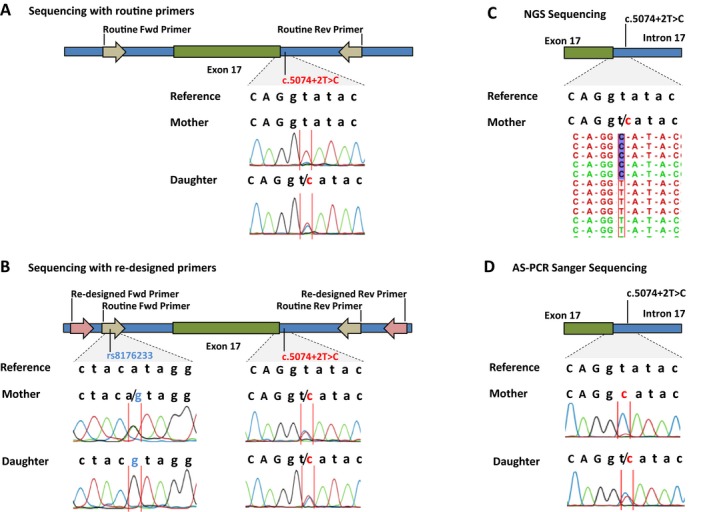
Analysis of a mother‐daughter dyad for the presence of the splice site mutation in *BRCA1* intron 17. (A) Small cytosine peak observed on the mother sample after several PCR protocols modifications and the heterozygous c.5074+2T>C (BRCA1 NM_007294.3) splice site mutation in the daughter. (B) A new set of primers detected a polymorphism (rs8176233) in the routine primers for exon 17 of *BRCA1*. The new primer pair was able to detect the splice site mutation in the mother in heterozygosity. (C) Ion PGM sequencing detected the index patient mutation in 50% of the reads. (D) Using allele specific primers (AS‐PCR), we confirmed that the mutant allele was on the same allele of the polymorphism (rs8176233) in the mother.

First, using the same DNA sample of the mother used in the original test, we carried out a thorough analysis of *BRCA1* exon 17 using different Taq‐polymerase enzymes and PCR conditions. This exhaustive analysis revealed in few capillary sequencing assays a small, almost imperceptible, electropherogram peak of the same mutated allele as found in her daughter (Fig. 1A). These data indicated that there was a reproducible preferential amplification of the normal allele rather than both alleles, most likely caused by a mismatch in the primer‐binding region.

In this sense, we designed a new set of primer pair for amplifying *BRCA1* exon 17, located 6nt upstream the routine forward primer and 95nt downstream the routine reverse primer. With this new set of primers, the mother was found to carry a polymorphism located in the 11th nucleotide from the 3′ end of the forward routine primer (c.4987‐92A>G, rs8176233, MAF 0.35) in heterozygous status, while the daughter was homozygous for the same polymorphism. Furthermore, this new set of primers confirmed that the mother was a heterozygous carrier of the splice site pathogenic mutation (c.5074+2T>C) (Fig. 1B). Concomitantly, we performed the complete sequencing of both *BRCA1* and *BRCA2* genes in a NGS platform (Ion Torrent‐PGM), which results also revealed the presence of the *BRCA1* mutation c.5074+2T>C in heterozygosity, with 50% of the reads presenting the mutated allele (Fig. 1C).

In order to prove that the rs8176233 SNP (c.4987‐92A>G) was in cis phase with the pathogenic mutation in the mother and caused the allele dropout, we performed AS‐PCR with primers sequences ending with the reference nucleotide A (adenine) and the polymorphic G (guanine) of the c.4987‐92A>G polymorphism. AS‐PCR using the G primer sequenced both alleles from the daughter, since both chromosomes carried the polymorphism (homozygous), showing the mutation c.5074+2T>C in heterozygosis. Conversely, the G primer sequenced only the mutant allele of the mother, showing the mutation c.5074+2T>C in apparently homozygosis (Fig. 1D), confirming that the mutation was on the same allele of the polymorphism.

## Discussion

Single‐nucleotide polymorphisms constitute the great majority of variations in the human genome (Lefever et al. 2013) and the advances in massive parallel sequencing projects resulted in a rapid increase in the SNP density described for the human genome. To date, the number of validated unique SNPs, small INDELs and multiple base nucleotide variations described at the dbSNP database has exceeded 100 million (dbSNP build 146), meaning that typically one in every 30 bases of the human genome presents a variation in the population.

**Figure 2:**
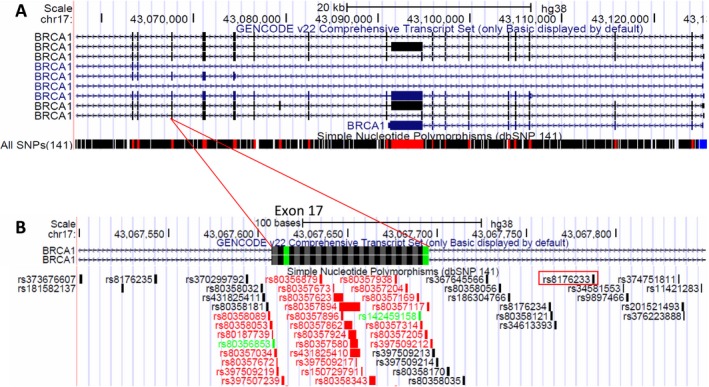
*BRCA1* SNP density. (A) Significant number of SNPs throughout the *BRCA1* gene. (B) SNPs throughout exon 17 area from *BRCA1* gene, where a SNP was found to affect the primer annealing (red rectangle).

Single‐nucleotide polymorphisms are nonuniformly dispersed throughout the genome, being much more frequent in noncoding regions, which are under less selective pressure than coding sequences (Fedorov et al. 2002; Kendal 2003). This issue is of great interest in genetic screening, since the intron sequences are the usual site for primer designing. In this sense, analyzing *BRCA1* exon 17 boundaries using the UCSC genome browser (https://genome.ucsc.edu/cgi-bin/hgGateway) allowed us to identify a significant number of SNPs upstream and downstream of exon 17, and almost in the entire gene (Fig. 2), making a proper SNP‐free primer designing a laborious process and almost impossible. Nevertheless, if SNP‐free primers cannot always be designed depending on the genomic region, an extra caution needs to be taken for avoiding SNPs with higher allele frequencies, such as the one we found in our study.

Concerning the clinical case here reported, the initial false negative result of the mother occurred because of a SNP identified in the middle of the primer sequence, and without the test of the daughter, we hardly would have found the pathogenic mutation. This raises the question of how frequent false negative genetic testing can be due to SNPs in primer‐annealing regions of any gene. To further illustrate this problem, we also present an additional case of a *HFE* gene mutation undisclosed because of a set of primers that encompassed a SNP in the middle of its sequence (Figure S1).

As exemplified in our two cases, allele dropout can occur when a variant is located in a primer binding site, preventing primer hybridization and leading to failed amplification and allele bias (Ikegawa et al. 2002). However, the effect of sequence specificity (i.e., mismatch type and position) of the primer is still poorly understood and dependent on sequence context and PCR conditions. Wu and colleagues reported that mismatches at the internal positions lead to PCR preferential amplification, whereas mismatches in the last 3 or 4 positions at the 3′ end shows minimal or no extension (Wu et al. 2009). In this report, our SNP was reported in the internal part of the primer (11th nucleotide from the primer 3′ end), suggesting that the probable explanation for lower amplification of the mutated allele was the preferential amplification of the wild‐type allele. The impairment of amplification, rather than a complete abrogation of primer binding, is also corroborated by the effective amplification observed in the daughter, who is homozygote for the rs8176233 SNP.

Another problem associated with PCR of diploid genomic DNA is the occasional amplification failure of one allele due to sequence independent factors. These include a variety of sampling and/or molecular events, such as variations in DNA extraction quantity or quality, presence of PCR inhibitors, variations in pipetting volumes of reagents or templates, imprecisions in thermocycler temperatures and so forth (Blais et al. 2015). Currently, the only large study to evaluate the frequency of allele dropout in a diagnostic setting is the one of Blais et al. (2015). In their set, the review of 30,769 patients genotypes revealed a rate of 0.3% of allele dropout events, where most of them (90%) were associated to nonreproducible PCR failures rather than sequence variants interfering with the assay, suggesting that careful primer design cannot prevent most of these errors (Blais et al. 2015).

In the case of NGS, allele dropout can be significantly reduced by multiple overlapping amplicons for each target, although this approach can significantly affect both the size and the cost of the targeted gene panel. In addition, analysis of individual amplicon dosage can be used to assess PCR allele dropout and amplicons that appear amplified from a single allele can be resequenced with other techniques (Judkins et al. 2015). Another alternative is the use of probe‐based target enrichment techniques that contain probes up to 120 nucleotides, which are much longer than typical PCR primers, and are usually not affected by single variants, reducing the chances of allele dropout.

Finally, considering that several PCR‐based approaches, including capillary sequencing and NGS, are extensively used in clinical diagnostics, the evidence presented here call attention for one of the limitations of genetic testing in general. The scarce data available on the literature, as well as the absence of large studies investigating the prevalence of this phenomenon, urge to the necessity of further investigation on this subject. Furthermore, this real‐life clinical case illustrate that in case of disagreement between the results of genetic testing and a strong clinical and familial history, it may be worthwhile to confirm the results using a second sequence approach to minimize the risk of a false negative result.

## Conflict of Interest

None declared.

## Supporting information


**Appendix S1.** Methods.
**Figure S1.** Presence of a SNP in primer‐annealing sequence interferes in the analysis of exon 2 of HFE gene.
