# Supplementary material for: Pitfalls in genetic testing: a case of a SNP in primer‐annealing region leading to allele dropout in BRCA1
Source: Mol Genet Genomic Med. 2017 May 11;5(4):443–7. doi: 10.1002/mgg3.295 (PMC5511807; doi:10.1002/mgg3.295)
Supplement: Supplementary file 1 — Appendix S1. Methods. Figure S1. Presence of a SNP in primer‐annealing sequence interferes in the analysis of exon 2 of HFE gene. [file MGG3-5-443-s001.doc]

**Pitfalls in *BRCA1* mutation screening: a case of a SNP in primer-annealing region leading to allele dropout**

**SUPPLEMENTARY INFORMATION**

**METHODS**

**Next Generation Sequencing**

Sequencing libraries were prepared from 10 ng of DNA samples using the Ion AmpliSeq™ BRCA1/2 Panel together with the Ion AmpliSeq^TM^ Library Preparation Kit (Thermo Fisher Scientific) according to the manufacturer’s guidelines. The libraries were quantified with the Qubit® 2.0 Fluorometer, using the Qubit® ds DNA HS assay kit. Templates were prepared and enriched using the Ion OneTouch^TM^ 2 system and the Ion PGM^TM^ Template OT2 200 Kit. Sequencing was performed with the Ion PGM^TM^ Sequencing 200Kit v2, the Ion 316v2 chip and the Ion Torrent PGM instrument. Sequencing reads were quality-filtered and sorted according to barcodes using Torrent Suite Browser 4.0.1 and mapped against the human genome reference (hg19). Sequencing parameters included 100% of the target region (entire *BRCA1/2* coding region and exon-intron boundaries) represented by the sequencing reads at a minimum mean coverage of 100X. SNVs and indels were identified using the combination of three approaches: the standard TorrentVariantCaller v4.0.r73742 plugin from Torrent Suite Browser; an in-house custom pipeline that uses the TorrentVariantCaller v4.0.5 and SnpEFF v4.1 (Cingolani et al, 2012) softwares; and CLC Genomic Workbench 6 software. For variant calling, the minimal coverage was set as 30X and the variant had to be present at a minimum frequency of 20%.

**
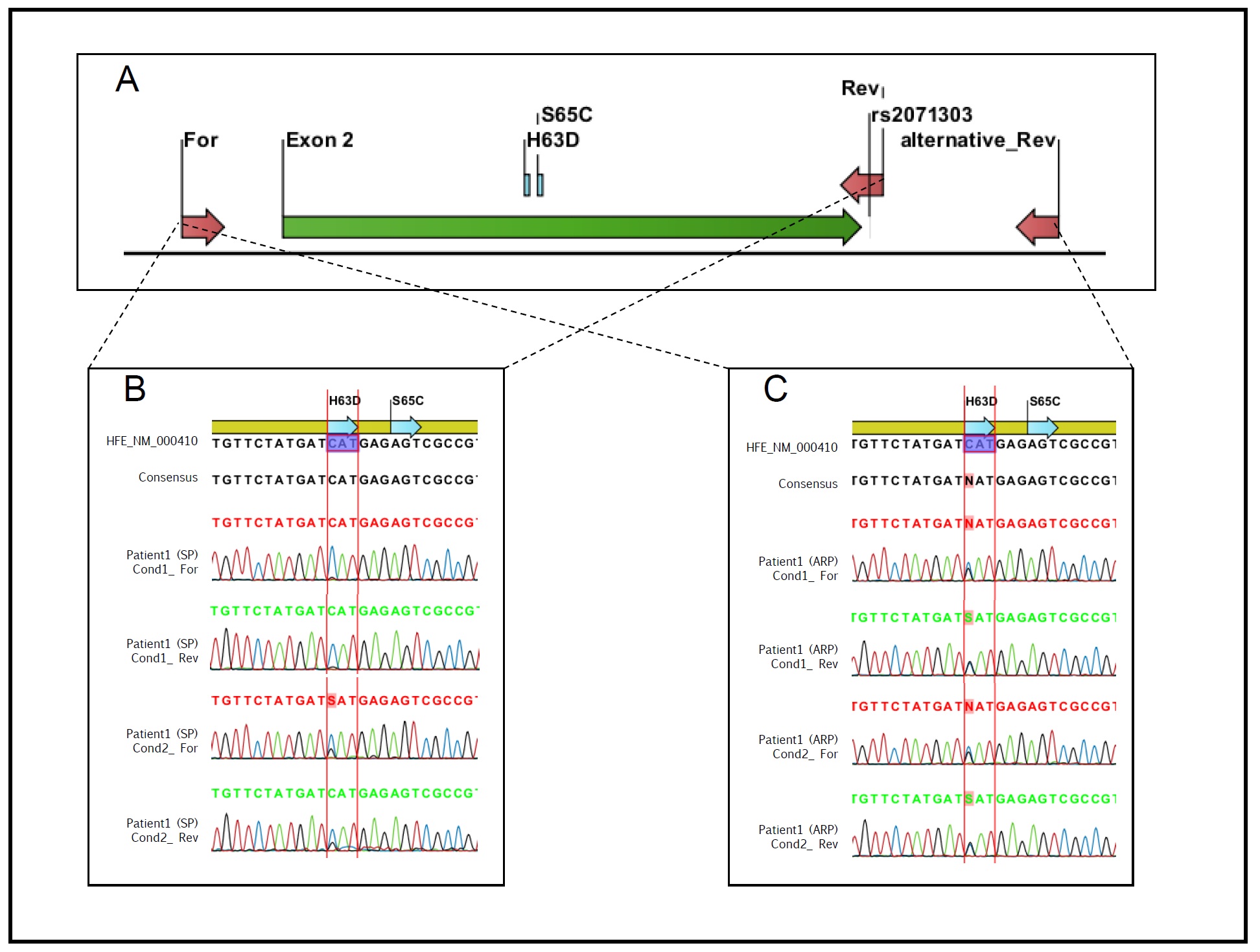
**

**Supplementary Figure 1: Presence of a SNP in primer-annealing sequence interferes in the analysis of exon 2 of HFE gene.** A) Schematic representation of exon 2 region of HFE gene, where the position of the standard forward and reverse primers (For and Rev) used to uncover two possible mutations (H63D and S65C) are specified. An alternative reverse primer (alternative rev) was designed after a more thorough analysis of primer-annealing regions revealed a polymorphism (rs2071303) that could impairs the precise sequencing of the exon. B) Using our previous standard set of primers (SP) and two different PCR conditions (Cond1 and Cond2), we identified a dubious guanine peak on a patient. This result prompted us to perform a database analysis of the primer-annealing regions and led to the uncovering of a SNP in the reverse primer region (rs2071303). C) Using an alternative reverse primer (ARP), we could elucidate the heterogeneity of the C>G alteration in our patient.

**REFERENCES**

Cingolani P, Platts A, Wang le L, Coon M, Nguyen T, Wang L, Land SJ, Lu X, Ruden DM. A program for annotating and predicting the effects of single nucleotide polymorphisms, SnpEff: SNPs in the genome of Drosophila melanogaster strain w1118; iso-2; iso-3. *Fly* (Austin). 2012 Apr-Jun;6(2):80-92.
